# Towards a Non-Human Primate Model of Alpha-Synucleinopathy for Development of Therapeutics for Parkinson’s Disease: Optimization of AAV1/2 Delivery Parameters to Drive Sustained Expression of Alpha Synuclein and Dopaminergic Degeneration in Macaque

**DOI:** 10.1371/journal.pone.0167235

**Published:** 2016-11-30

**Authors:** James B. Koprich, Tom H. Johnston, Gabriela Reyes, Vanessa Omana, Jonathan M. Brotchie

**Affiliations:** 1 Atuka Inc., Toronto, Canada; 2 Krembil Research Institute, University Health Network, Toronto, Canada; Emory University, UNITED STATES

## Abstract

Recent failures in clinical trials for disease modification in Parkinson’s disease have highlighted the need for a non-human primate model of the synucleinopathy underpinning dopaminergic neuron degeneration. The present study was defined to begin the development of such a model in cynomolgus macaque. We have validated surgical and vector parameters to define a means to provide a robust over-expression of alpha-synuclein which is associated with Lewy-like pathology and robust degeneration of the nigrostriatal pathway. Thus, an AAV1/2 vector incorporating strong transcription and transduction regulatory elements was used to deliver the gene for the human A53T mutation of alpha-synuclein. When injected into 4 sites within each substantia nigra (7 μl per site, 1.7 x 10^12^ gp/ml), this vector provided expression lasting at least 4 months, and a 50% loss of nigral dopaminergic neurons and a 60% reduction in striatal dopamine. Further studies will be required to develop this methodology into a validated model of value as a drug development platform.

## Introduction

Evidence continues to support that Parkinson’s disease (PD) is an alpha-synucleinopathy [1–3]. Irrespective of etiology and varying upstream mechanisms, deposition of alpha-synuclein (aSyn) is a defining pathological feature of PD and is increasingly being understood to be involved in synaptic dysfunction [4–6] and axonal transport deficits [7, 8] and produces ER-Golgi stress [9, 10], to name a few key cell biological processes. Accumulation of aSyn has been found upon postmortem examination in the olfactory bulb, amygdala, nucleus basalis, substantia nigra, locus coeruleus, dorsal motor nucleus of vagus and the cerebral cortex [11, 12]. It has recently been shown that aSyn accumulations are more widely spread throughout the brain and occur earlier than previously understood [13]. While the identification of Lewy bodies/ neurites is part of the final diagnosis of PD, evidence supports that earlier forms or strains of aSyn as the toxic culprits [14] and that the Lewy bodies may serve more as a marker of ongoing aSyn based dysfunction but likely does not represent the aSyn actively involved in cellular dysfunction.

With widespread aSyn deposition, and resulting dysfunction, drug development programs need to consider whether their targets continue to be engagable against this background. This concept is highlighted by the recently failed clinical trials for neurturin and PYM50028 both of which putatively affording neuroprotection through GDNF signaling, and both of which showed neuroprotective benefits in toxin-based models of PD in rodents and NHP. It is now clear from rat studies that the receptor for GDNF, ret, is downregulated under conditions of aSyn overexpression and thus lack target engagement may have contributed to lack of demonstrable clinical efficacy [15]. Thus, demonstrating neuroprotection in a toxin based model of PD (e.g. 6-OHDA or MPTP) is thus no longer sufficiently compelling evidence of potential efficacy to support advancing a drug candidate to clinical development as a disease modifying therapy in PD. To this end PD models based on overexpression of aSyn have been developed and are being utilized in therapeutic development.

Models based on overexpression of aSyn ranging from yeast, cell lines and *C*. *elegans* to rodents and primate have been developed and have been used in their respective places in the drug development process. Primate models have been described showing, proof of concept, that viral vector delivery of aSyn can achieve expression in target populations of neurons and result in degeneration [16, 17]. However, the degree of variability reported is not consistent with a drug development platform that could be routinely implemented. Thus, a power calculation based upon reported deficit in dopamine neurons and variability, would define a prohibitive number of animals per group to demonstrate a drug effect reducing degeneration by 30%. In an attempt to reduce this variability, we have chosen to employ an AAV vector with a stronger promoter than those previously reported. These previous studies, employed marmosets as their primate species. In the current study, we have elected to define parameters for delivering an AAV-aSyn vector in cynomolgus macaques. From a drug development perspective, the macaque is preferred as it is macaque that the majority of non-human primate pharmacokinetic, safety and toxicology studies are performed. Macaque also offers opportunities, not afforded in smaller species, for imaging components, these can be especially useful in incorporating target engagement studies into a development program progressing from NHP to the clinic.

The current study aimed to define the parameters for developing an aSyn-driven model in the cynomolgus macaque. The approach was based upon the stereotactic delivery of an adeno-associated viral (AAV) vector incorporating the gene for the A53T mutant form of human aSyn (hA53T-aSyn). Surgical targeting, volumes and titer of vector delivered were considered with the goal of producing > 85% transduction of dopaminergic neurons in the target structure, the substantia nigra, obtaining expression and accumulation of aSyn that was sustained over a period of at least 4 months, transport of alpha-synuclein transgene to the striatum and evidence of a resulting degeneration of the nigrostriatal DA system.

## Methods

### Animals and husbandry

Six, 8 years old, female cynomolgus macaques were included in the study. Animals were obtained from Grand Forest Ltd., Guangxi, PRC. Animals were group housed, in caging exceeding NIH, CCAC and EU guidelines, and provided with an enrichment program and fresh fruit along with their dry diet and water freely available. All husbandry, housing and experimental procedures were conducted at WinCON TheraCell Biotechnologies Co. Ltd. (Nanning, PRC) and under an IACUC animal use protocol specific for this study (W00023) and approved by WinCON TheraCell Biotechnologies Co. Ltd.

### Viral vectors

Adeno associated vectors (AAV) of a 1/2 serotype were designed such that expression was driven by a chicken beta actin (CBA) promoter hybridized with the cytomegalovirus (CMV) immediate early enhancer sequence. In addition, a woodchuck post-transcriptional regulatory element (WPRE) and a bovine growth hormone polyadenylation sequence (bGH-polyA) were incorporated to further enhance transcription following transduction. AAV1/2 is a chimeric vector where the capsid expresses AAV1 and AAV2 serotype proteins in a 1:1 ratio and uses the inverted terminal repeats (ITRs) from AAV2 according to the following scheme: CMV/CBA promoter—-human A53T alpha-synuclein or GFP or scrambled human A53T alpha-synuclein—-WPRE-bGH-polyA—-ITR [18]. The vectors were produced by GeneDetect Ltd., Auckland, New Zealand. Viral titers were determined by quantitative PCR (Applied Biosystems 7900 QPCR) with primers directed to the WPRE region, thus representing the number of functional physical particles of AAV in the solution containing the genome to be delivered. Full details of hA53T aSyn and green fluorescent protein (GFP) vector design can be found in Koprich et al., 2010 [19]. The scrambled and non-scrambled human A53T alpha-synuclein was tagged with hemaglutannin (HA) on the carboxy terminal end. The scrambled sequence has previously been used [20].

### Surgery/MRI

Anatomical MRI (magnetic resonance imaging) was performed on each animal in order to derive surgical coordinates and was conducted under ketamine anesthesia (15 mg/kg ketamine combined with 0.04 mg/kg atropine, IM) and maintained with isofluorane while spontaneously breathing. Animals were placed into a MRI compatible stereotaxic frame (Crist Instruments, Hagerstown, MD) and MRI (3T, General Electric) was performed to obtain T1 weighted axial slices for future surgical targeting.

The surgical procedure was performed under endotracheal isoflurane anesthesia in sterile conditions with oversight of veterinary medicine staff. All cranial injections were performed with the animal in a stereotactic head holder. Precise stereotaxic coordinates for all surgeries were calculated from the MRI of each individual monkey. Following cranial preparation, an incision was made over the target area and skin, muscle and fascia were retracted to expose the cranial surface. Bilateral burr holes were made over the target areas. A small incision was made in the dura mater above the desired injection sites and the tip of a Hamilton syringe (26G) was lowered to the desired site for microinjection. Injections containing the AAV1/2-alpha-synuclein or control vector were made at a speed of 2 uL/min and a volume of 5 or 7 μl each into 4 sites in the unilateral substantia nigra and then repeated on the contralateral side. The needle was maintained in place for an additional 5 min after injection before being withdrawn slowly and moved to different sites. Following the injection, the exposed dura was covered with Gelfoam and the incision closed with an interrupted 6–0 monofilament suture. Monkeys received an IV antibiotic treatment during surgery and orally thereafter 2x/day. Postoperatively, monkeys received meloxicam (0.1 mg/kg, p.o., 1/day for 5 days) and tramadol (3 mg/kg, i.m., 2/day for 3 days) for analgesia management. The animals were carefully monitored during the post-operative period.

### Necropsy

At the end of the study (17 weeks post AAV delivery), all animals were euthanized by pentobarbital overdose, thus, anesthesia was induced with ketamine (15mg/kg) followed by a terminal dose of pentobarbital (100 mg/kg, i.v.) and then perfused, transcardially, with 750 mL of room temperature 0.1% heparinised 0.9% saline followed by 750 mL of ice-cold 0.9% saline. The brain was then carefully removed from the calvaria and blocked into 2–4 mm coronal slabs and prepared for subsequent analyses.

### HPLC

Striatal samples were freshly dissected from a 4 mm slab using a 2 mm punch and were homogenized in 200 ul of 0.1M TCA, which contained 10^−2^ M sodium acetate, 10–4 M EDTA and 10.5% methanol (pH 3.8) using a tissue dismembrator (Fisher Scientific). Samples were centrifuged at 10,000 x g for 20 minutes at 4°C. The supernatant was removed and stored at –80°C. The pellet was used for total protein content analysis. Supernatant was thawed and centrifuged at 10,000 x g for 20 minutes at 4°C. Dopamine, homovanillic acid and 3,4-dihydroxyphenylacetic acid levels were determined by a specific HPLC assay utilizing an Antec Decade II (oxidation: 0.5) electrochemical detector operated at 33°C. Samples of the supernatant were injected using a Waters 717+ autosampler onto a Phenomenex Nucleosil (5u, 100A) C18 HPLC column (150 x 4.60 mm). Analytes were eluted with a mobile phase consisting of 89.5% 0.1M TCA, 10^−2^ M sodium acetate, 10^−4^ M EDTA and 10.5% methanol (pH 3.8). Solvent was delivered at 0.8 ml/min using a Waters 515 HPLC pump. Using this HPLC solvent, analytes were observed in the following order: DOPAC, dopamine, and HVA. HPLC control and data acquisition were managed by Waters Empower software. Total protein for each sample was determined using the Peirce BCA protein assay (BCA assay, Pierce, Rockford, IL). Values of catecholamines are expressed as ng analyte/ mg total protein [19, 21].

### ELISA

Putamen samples were freshly dissected from a 4 mm tissue slab using a 2 mm punch. Homogenization was performed using cold RIPA buffer (Biobasic Canada, Inc., Ontario) supplemented with 1 X protease inhibitor cocktail (Roche, Indianapolis, IN), followed by the addition of 8.2M Guanidine HCL/ 82mM Tris-HCL (pH 8.0) to yield a solution with 5M final guanidine concentration. Samples were sonicated (QSonica, Newtown, CT) at 70% for 10 seconds each time and immediately placed back on ice. The homogenates were set in a rotating shaker to mix for 3 hours at room temperature then centrifuged at 16,000 x g for 20 minutes at 4°C (Labnet International, Inc., Edison, NJ). The supernatant was aliquoted and frozen at -80°C. The BCA Protein Assay was used to measure (A562nm) total protein concentration from supernatant (Pierce, Thermo Scientific, Rockford, IL). The levels of α-synuclein were determined by Human α-Synuclein ELISA kit (Invitrogen, Carlsbad, CA) according to the manufacturer’s instructions [8, 22].

### Dopamine transporter autoradiography

Levels of striatal dopamine transporter (DAT) binding were assessed by [^125^I]-RTI-121 binding autoradiography in sections prepared from fresh-frozen forebrain tissue. Briefly, thawed slides (two slides per animal) were placed in 0.1M Dulbecco’s PBS, pH 7.4 for 30 min, room temperature, with gentle agitation. To determine total binding, sections were then placed in the same buffer containing 35 pM [^125^I]-RTI-121 (Perkin-Elmer, Bridgeville, PA, U.S.A.; specific activity 2200 Ci/μmol) for 90 min at room temperature. To determine non-specific binding, sections were incubated in buffer containing 35 pM [^125^I]-RTI-121 and 100 μM GBR 12909 (Tocris Bioscience, Ellisville, MO, U.S.A.). All slides were then washed (2 x 20 min) in ice-cold 0.1M Dulbecco’s PBS, pH 7.4, dipped 10 sec in ice-cold distilled water and air-dried. Slides were then apposed to autoradiographic film (Biomax MR, Eastman Kodak Company, Rochester, NY, U.S.A.), together with [^14^C]-microscale standards (GE Healthcare, Piscataway, NJ, U.S.A.), and left for 2 days at room temperature before developing. Autoradiograms were analysed using MCID software (Image Research Inc, St. Catharines, ON, Canada). Densitometric analysis of 2 striata from each animal was carried out whereby a reference curve of c.p.m. *versus* optical density was calculated from β-emitting [^14^C] microscale standards and used to quantify the intensity of signal as nCi/g. Background intensity was subtracted from each reading. Data were then expressed as mean ± s.e.m. signal intensity for each treatment group. Non-specific binding was calculated in the same way and subtracted from the total to give specific binding [19, 23].

### Histology and stereology

Cryo-protected sections of post-fixed mesencephalon were processed for immunohistochemistry. Brains were cut frozen in the coronal plane at a thickness of 40 μm on a sledge microtome (Leica, SM2000R) resulting in twelve series of sections stored in cryoprotectant. Using the biotinylated antibody method, two one-twelfth series of sections were processed for visualization of tyrosine hydroxylase (TH) and alpha synuclein (LB509). Another one-twelfth series of sections were processed for co-visualization of TH, alpha synuclein, and human influenza hemagglutinin (HA) via immunofluorescence. Details below.

### Tyrosine hydroxylase immunohistochemistry

Free floating sections were went through several washes in a PBS solution containing 0.5% Tween-20, endogenous peroxidase was quenched in a 0.6% hydrogen peroxide solution and background staining inhibited in a blocking solution containing 10% normal goat serum (NGS) and 2% bovine serum albumin (BSA). Tissue was then incubated overnight in rabbit anti-TH antibody (1:5000; Chemicon, AB152), followed by three washes in PBS, then incubated 2 hours in biotinylated IgG antibody (goat anti-rabbit 1:500, Jackson Immuno, West Grove, PA, Cat. #111-065-144), three more washes in PBS, and incubated 1 hour in avidin-biotin complex (Vectastain Elite ABC Kit; Vector, Cat. #PK-6100). TH immunostaining was visualized using 3,3-diaminobenzidine (ImmPACT DAB, Vector, Cat. # PK-4105).). Sections were then mounted on glass slides, allowed to dry, dipped into dH_2_0, dehydrated through graded alcohols, clarified in xylenes, and coverslipped using DePex mounting media (VWR, product 361254D).

### Alpha Synuclein Immunohistochemistry

Alpha synuclein was visualized in separate one-twelfth series using either antibodies raised against the LB509 clone. Briefly, following several washes in a PBS solution containing 0.5% Triton X-100, endogenous peroxidase was quenched in a 0.6% hydrogen peroxide solution and background staining inhibited in a blocking solution containing 10% NGS and 2% BSA. Tissue treated for LB509 visualization was incubated overnight in mouse anti-alpha synuclein (LB509 clone) antibody (1:200; Invitrogen, Cat. #18–0215), followed by three washes in PBS, then incubated 2 hours in biotinylated IgG antibody (goat anti-mouse 1:400; Vector, Cat. # BA-9200), three more washes in PBS, and incubated 1 hour in avidin-biotin complex (Vectastain Elite ABC Kit; Vector, Cat. #PK-6100). Alpha synuclein was visualized using 3,3-diaminobenzidine (ImmPACT DAB, Vector, Cat. # PK-4105). Sections were then mounted on glass slides, allowed to dry, dipped into dH_2_0, dehydrated through graded alcohols, clarified in xylenes, and coverslipped using DePex mounting media (VWR, product 361254D).

### Immunofluorescence of tyrosine hydroxylase, hemagglutinin and alpha-synuclein

One-twelfth series of sections were washed several times in a PBS solution containing 0.1% Tween-20, then background staining was inhibited in a blocking solution containing 10% normal donkey serum (NDS) and 2% bovine serum albumin (BSA). Tissue was then incubated overnight in a solution containing rabbit anti-TH antibody (1:1000; Pel-Freez, code 40101–0), mouse anti-human alpha synuclein (1:500; Invitrogen, Cat. # 32–8100) and goat anti-HA (1:500; Abcam, AB9134). Following three washes in PBS, the sections were incubated 2 hours in PBS with 0.1% Tween-20 and 2% NDS, and containing Alexa Fluor donkey anti-rabbit 568 (1:500; Invitrogen A10042), Alexa Fluor donkey anti-mouse 488 (1:500; Invitrogen A21202), and Alexa Fluor donkey anti-goat 647 (1:500; Invitrogen A214447), protected from light. Sections were washed several time in PBS, mounted on glass slides, allowed to dry, dipped into dH_2_0, and coverslipped using aqueous fluorescence mounting media (Dako, code 3023). Slides were stored protected from light at 4°C.

### Stereological Estimation of Nigral Tyrosine Hydroxylase Immunoreactive Cell Population

Estimates of nigral TH^+ve^ and aSyn^+ve^ neuronal populations were performed using Stereo Investigator software (MBF Bioscience, Williston, VT, U.S.A.) according to stereological principles. For double label counts to determine the ratio of SN TH^+ve^ neurons overexpressing aSyn, detection was set above that of control tissue (representing endogenous signal) and applied to tissue exposed to viral vector produced A53T aSyn. Eleven coronal sections, each separated by 480 μm, from anterior to posterior SN, were used for counting. Stereology was performed using a Zeiss AxioImager M2 microscope coupled to a colour digital camera or monochrome camera (fluorescent images) for visualization of tissue sections. Total numbers of positive neurons were estimated from coded slides using the optical fractionator method. For each tissue section analyzed, section thickness was assessed empirically with guard zones of 2μm and counts performed in a counting grid size of 250 μm x 250 μm, systematic random sampling (SRS) frame of 1000 μm x 750 μm and probe dissector height of 20μm. Coefficients of error (Gunderson, m = 1) were calculated by the Stereo Investigator software and values <0.10 were accepted.

### Statistics

In Experiment 1, groups were compared on each dependent measure using a one-tailed t-test with P set at <0.05 to be considered significant. In Experiment 2, means were calculated and percent difference from control were presented without the use of statistics since only 2 cases were included per group.

## Results

### Experiment 1

#### Transgene expression

In the first experiment, 3 female cynomolgus macaques (8 yrs old) received injections of AAV1/2-hA53T-aSyn and AAV1/2-GFP into the substantia nigra. In each animal, one hemisphere received AAV1/2-hA53T-aSyn and the other AAV1/2-GFP, thus 3 hemispheres for each injected AAV1/2 constituted the sample sizes for Experiment 1. The amount and concentration of viral vector injected was 20 μl (over 4 sites in each hemisphere, 5 μl/site) and 1.7 x 10^12^ gp/ml, respectively. Animals were euthanized 17 weeks later.

**Fig 1:**
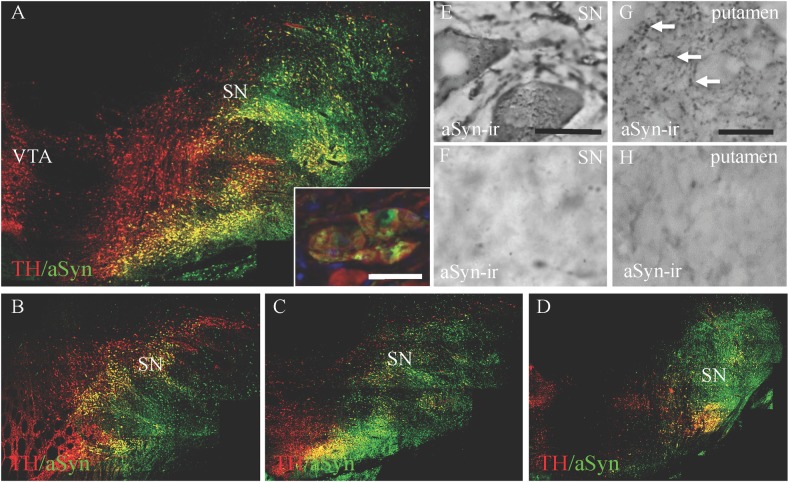
Transgene expression in the substantia nigra and putamen 17 weeks post surgical delivery of AAV1/2 hA53T alpha synuclein. Panel A shows wide spread overexpression of alpha synuclein (aSyn) in dopamine neurons within the macaque substantia nigra (SN). This was evident throughout the anatomical boundaries of the SN (Panels A-D). The inset of Panel A and Panel E shows high power magnification of the accumulation of aSyn in SN cell bodies. Transport and expression of aSyn was observed throughout the putamen in animals that received SN injections of AAV1/2 hA53T alpha synuclein (Panel G) compared to endogenous expression of aSyn in the SN (Panel F) and putamen (Panel H) of AAV1/2 GFP injected controls. Scale bars: in inset of panel A, 50 μm; in panel E, 25 μm and in panel F-H, 50 μm.

Overexpression of aSyn could be seen throughout the extent of the SN (Fig 1A–1D). Double label immunofluorescence showed that the majority of SN dopamine neurons were infected (mean ± SEM: 86% ±10.6) by the vector and were expressing the transgene. Higher magnification showed that the expression of aSyn in the dopamine neurons took on an aggregated or accumulated appearance rather than a diffuse pattern (inset of Fig 1A). Using immunohistochemistry, aSyn immunoreactivity (ir) in the hemispheres exposed to AAV1/2 hA53T-aSyn revealed dystrophic neurites (putamen) and apparently unhealthy SN neurons (Fig 1E and 1G) while, in contrast, aSyn-ir in hemispheres receiving AAV1/2-GFP was minimal in both the SN and the putamen, revealing endogenous reactivity (Fig 1F and 1H). Over-expression of aSyn was evident in the caudate (data not shown) and the putamen when comparing GFP to aSyn exposed cases (Fig 1E vs. 1G).

Alpha-synuclein levels, quantified in the putamen by ELISA, were found to be 34% higher in animals that received AAV1/2 A53T aSyn (mean ±SEM: 4.68 μg/μg protein ±1.03) compared to control (mean ±SEM: 3.07 μg/μg protein ±0.66).

#### Lesion assessment

**Fig 2:**
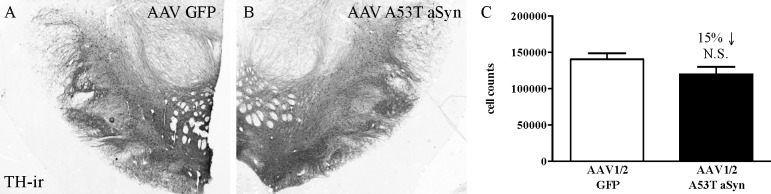
No significant loss of substantia nigra dopamine neurons. Cell counting stereology was performed on tyrosine hydroxylase immunoreactive neurons within the borders of the substantia nigra from animals exposed to 17 weeks of either AAV1/2 driven overexpression of human A53T alpha-synuclein (A) or GFP (B) (both 20 μl of 1.7 x 10^12^ gp/ml). No significant difference in total estimated numbers was observed, while a 15% reduction between the groups was calculated (P>0.05, NS) (C, mean ±SEM).

**Fig 3:**
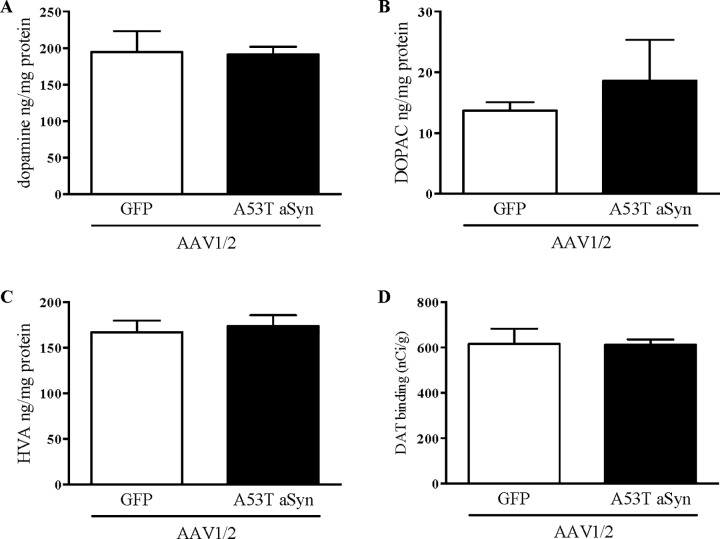
No changes on measures of dopamine neurochemistry or dopamine transporter levels in the putamen. Seventeen weeks following AAV1/2 delivery of hA53T alpha-synuclein or GFP (20 μl of 1.7 x 10^12^ gp/ml) to the macaque substantia nigra, levels of dopamine and its metabolites (3,4-Dihydroxyphenylacetic acid; DOPAC and homovanillic acid; HVA) were quantified by HPLC (A-C, mean ±SEM). No significant changes were observed on either measure. Dopamine transporter levels were determined by autoradiography and also showed no significant differences between groups (D, mean ±SEM).

Lesion extent was assessed by counting the number of dopaminergic neurons in the SN and by quantifying levels of putamenal DA and DAT. Cell counting stereology was conducted to estimate SN dopaminergic neuron numbers represented by TH immuno staining. No significant difference was seen between AAV-hA53T aSyn exposed hemispheres compared to AAV1/2-GFP exposed controls (t[4] = 1.57, P>0.05), although cell number was 15% lower in AAV1/2-hA53T exposed hemispheres (Fig 2). Similarly, no significant difference was seen between groups on measures of putamenal DA (t[4] = 0.12, P>0.05), DOPAC (t[4] = 0.72, P>0.05), HVA (t[4] = 0.39, P>0.05) or DAT (t[4] = 0.06, P>0.05) (all Fig 3).

### Experiment 2

#### Transgene expression

**Fig 4:**
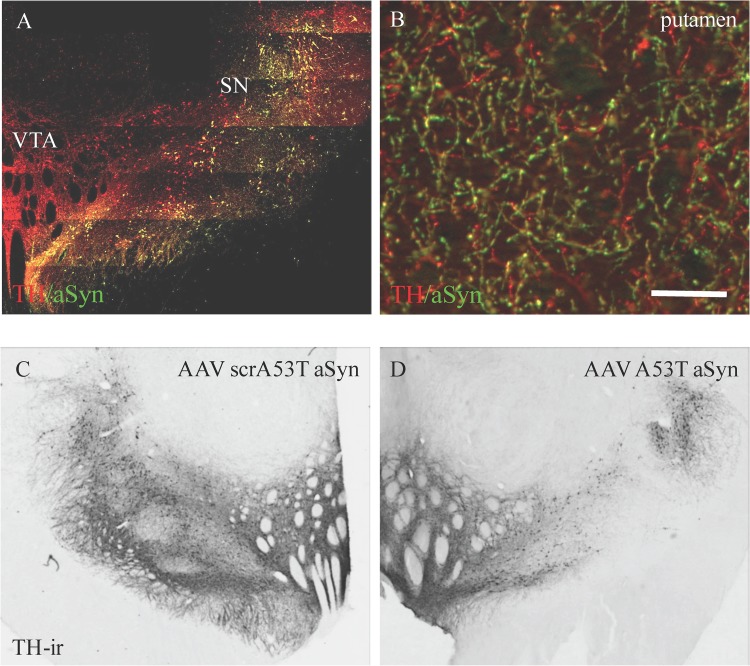
Higher volume of AAV1/2 hA53T alpha synuclein produces sustained transgene expression in the substantia nigra (SN) and putamen and produces a loss in SN dopamine neurons. In the second experiment AAV1/2 hA53T alpha synuclein was delivered either at the same titer (1.7 x 10^12^ gp/ml) and at higher volume (28 μl) or at the same volume (20 μl) and at a higher titer (5.1 x 10^12^ gp/ml) compared to Experiment 1. In both conditions, a loss in tyrosine hydroxylase immunoreactive neurons within the substantia nigra was observed (C vs. D and see Table 1). Despite the loss in these neurons, those that remained continued to express the transgene (A). Furthermore, overexpression of alpha synuclein remained elevated in the terminals of the putamen (B). The control used in Experiment 2 was scrambled hA53T alpha synuclein. Scale bar in panel B, 25 μm.

In Experiment 2, hemispheres of 3 animals were exposed to either higher titers (5.1 x 10^12^ gp/ml vs. 1.7 x 10^12^ gp/ml) of AAV1/2 (hA53T aSyn or scrambled hA53T as the control) or larger volumes (7 μl vs. 5 μl; N = 2 per condition) compared to Experiment 1, in order to define a dose and volume of vector that continued to cover the region of interest (SN), was expressed within SN dopamine neurons and additionally produced neurodegeneration in terms of nigrostriatal dopaminergic deficits. Animals were euthanized 17 weeks following viral vector delivery. Similar to Experiment 1, overexpression of aSyn in both the SN and the striatum was widespread (>85%) and within the region of interest (Fig 4A and 4B). Accumulations of aSyn could be seen within SN dopamine neurons as represented by TH immune staining (inset of Fig 4A).

Alpha synuclein levels, quantified in the putamen by ELISA, were 21 and 67% higher in hemispheres that received high titer/ low volume and low titer/ high volume AAV1/2 A53T aSyn, respectively, compared to control (Table 1).

**Table 1:** Summary of results from Experiment 2.

		stereology (SN)	putaminal HPLC (ng/mg protein)					putaminal DAT (nCi/g)	putaminal aSyn
condition		TH cell counts	DA	HVA	DOPAC	HVA/DA	DOPAC/DA		(ug/mg protein)
control	mean	133,717.50	92.95	99.94	5.74	1.09	0.07	657	3.08
high titer/low volume	mean	66,630.50	26.14	61.89	2.08	6.07	0.10	202.17	3.72
	*% of cntrl*	*50*	*28*	*62*	*36*	*558*	*152*	*31*	*121*
low titer/high volume	mean	68,123.50	36.96	78.52	2.18	3.14	0.07	414.03	5.14
	*% of cntrl*	*51*	*40*	*79*	*38*	*289*	*101*	*63*	*167*

control, scrA53T aSyn, 5.1 x 10^12^ gp/ml, 7 μl x 4 sites; high titer, 5.1 x 10^12^ gp/ml

low titer, 1.7 x 10^12^ gp/ml; high volume, 28 μl (7 μl x 4 sites); low volume, 20 μl (5 μl x 4 sites); Raw data of the results from Experiments 1 and 2 can be found in S1 Table.

#### Lesion assessment

Degree of lesion was assessed by quantifying endpoints related to nigrostriatal dopaminergic function (Table 1). Estimation of dopaminergic neuron number in the SN was performed by cell counting stereology of TH+ profiles. Hemispheres receiving the high titer/ low volume condition showed a 50% reduction in cell numbers and animals in the low titer/ high volume condition showed a 49% reduction in cell numbers, both compared to empty vector controls. On measures of putamenal dopamine neurochemistry, DA, DOPAC and HVA, hemispheres in the high titer/ low volume condition showed a 72%, 64% and 38% reduction, respectively, compared to controls. Hemispheres in the low titer/ high volume condition showed a 60%, 62% and 21% reduction, in DA, DOPAC and HVA, respectively, compared to controls. DAT levels were reduced in both conditions, by 70% in the high titer/ low volume condition and by 37% in the low titer/ high volume condition, compared to controls.

## Discussion

The current studies were designed to assess the potential for AAV1/2 to overexpress human A53T aSyn, and drive neurodegeneration, within the nigrostriatal tract of cynomolgus macaques as measured 17 weeks following surgical delivery. We show that AAV1/2 with CBA/CMV promoters and transduction regulation provides sustained transgene expression over this period of time in both SN dopamine neurons and their projections terminating in the striatum. After examining 3 combinations of viral titers and volumes of viral vector delivered, we identified combinations that can deliver sufficient transgene to produce evidence of degeneration.

In Experiment 1, 20 μl of 1.7 x 10^12^ gp/ml AAV1/2-hA53T-ASyn,distributed over 4 sites, 5 μl/site in the SN of one hemisphere. Expression covered >85% of SN neurons, was sustained for the duration of the exposure, accumulation of A53T aSyn was observed within SN dopamine neurons and fibers showed dense accumulation of A53T aSyn revealing a dystrophic morphology. However, this was not sufficient to produce significant reductions in neurons expressing TH in the SN, dopamine levels in the putamen nor in putaminal DAT binding compared to AAV1/2 GFP injected controls. These data demonstrated the utility of the surgical targeting strategy, that the AAV1/2 vector can drive expression of transgene for at least 4 months and that the aSyn produced by the transgene is transported to the striatum.

Experiment 2 was designed to deliver more viral particles, in order to expose the nigrostriatal system to hA53T-aSyn at a level that would produce deficits on measures of dopaminergic activity. This was achieved by either by increasing the volume of AAV1/2 delivered at the same titer as Experiment 1 (28 μl of 1.7 x 10^12^ gp/ml) or by increasing the titer of AAV1/2 delivered at the same volume as Experiment 1 (20 μl of 5.1 x 10^12^ gp/ml), in both conditions the volumes were divided equally over 4 sites in the SN of each hemisphere. Both strategies were sufficient to increase vector production of A53T aSyn produced deficits on all measures of DA function assessed, compared to controls. The levels of aSyn in the putamen were higher in Experiment 1 compared to Experiment 2 despite animals in Experiment 2 having had more viral particles delivered to the SN. This is likely due to differences in the amount of degeneration between the two Experiments. No significant loss in putaminal DA or DAT was shown in Experiment 1, while Experiment 2 did show significant losses on these measures. Since the dopaminergic fibers of the nigrostriatal pathway carry the transgene, a significant loss in these fibers could account for the disparate aSyn levels observed.

These data thus support, and extend to macaque, the findings of degeneration and lasting aSyn expression in two previous studies exposing the primate brain to aSyn by an AAV viral vector, where Kirik et al (2003) [17] and Eslamboli et al (2007) [16] delivered AAV2 and AAV2/5, respectively to the marmoset SN. In the Kirik et al study (2003), AAV2 delivery of wild type aSyn (8.2 x 10^11^ infectious units /ml; 2 x 3 μl injections) or A53T aSyn (1.4 x 10^12^ infectious units /ml; 2 x 3 μl injections) produced 30–60% SN dopamine neuron loss following 16 weeks of exposure. This is line with the timeframe and amount of cell loss observed in the current study. In a follow-up study using AAV2/5, Eslamboli et al (2007), showed that both wild type (2.1 x 10^13^ genome copies /ml; 2 x 2 μl injections) and A53T (4.9 x 10^13^ genome copies /ml; 2 x 2 μl injections) aSyn overexpression in marmosets produced variable SN dopamine neuron loss in the wild type condition (two out of 8 cases; 40% loss) while the A53T exposed primates showed consistent losses across all cases (>40% cell loss in 8 out of 9 cases). Again, these results are in line with those reported here. The results of Eslamboli et al (2007) provided rationale to use the A53T mutant in the current study.

In the present study, we focused on delivery of hA53T-aSyn in 8-year old animals. The pathology we describe is consistent with a recent report, Yang et al (2015) [24], in 3 rhesus macaques of different ages, 2, 8 and 22 years old, where lentiviral delivery of A53T aSyn to the SN produced Lewy neurites and accumulation of aSyn that was more abundant with the increasing age of the primate, after 8 weeks of exposure.

Importantly, the remaining nigrostriatal system continues to express the transgene allowing for potentially dysfunctional substrate to be examined for therapeutic efficacy and to evaluate target engagement (i.e. aSyn clearing compounds, neuroprotective pathways).

We found no significant difference between neurodegeneration driven by the low volume/ high titer condition compared to high volume/ low titer. For the purposes of further developing this model we will employ the high volume/ low titer combination to minimize vector requirement.

Preclinical drug development programs in PD based on therapeutics directly influencing aSyn or designed to show disease modification require a primate model. The methodology described here is the first step towards developing and validating such a model of PD. In particular, we see a need to define the timecourse of the degenerative process, the level of aSyn expression at longer timepoints, the potential for the degenerative process to elicit a parkinsonian behavioural phenotype, the molecular characteristics of the aSyn aggregations and the variability across larger group sizes.

## Supporting Information

S1 Table Raw data from Experiments 1 and 2. (JPG)
