# Supplementary figures and images for: Towards a Non-Human Primate Model of Alpha-Synucleinopathy for Development of Therapeutics for Parkinson’s Disease: Optimization of AAV1/2 Delivery Parameters to Drive Sustained Expression of Alpha Synuclein and Dopaminergic Degeneration in Macaque
Source: PLoS One. 2016 Nov 30;11(11):e0167235. doi: 10.1371/journal.pone.0167235 (PMC5130249; doi:10.1371/journal.pone.0167235)

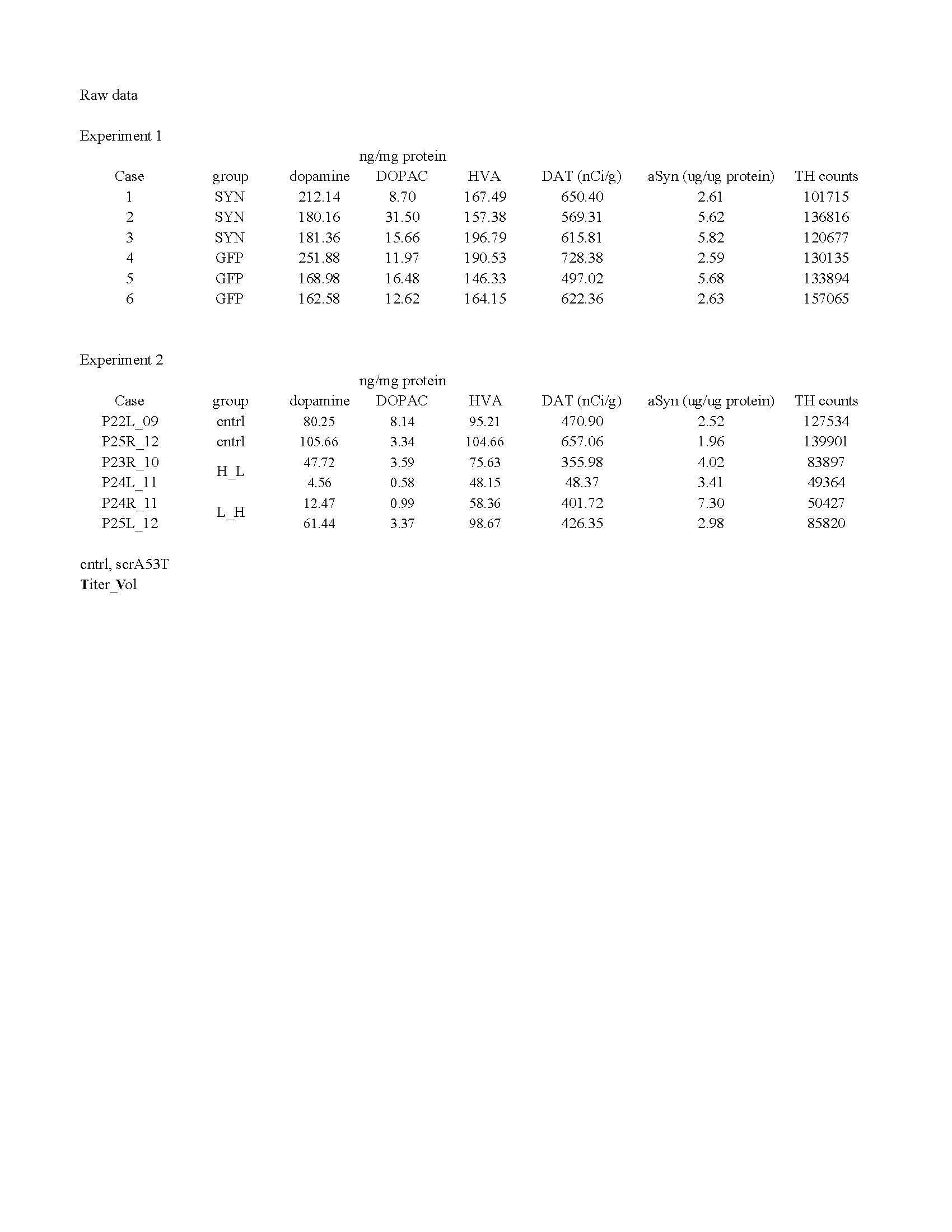

Supplement: S1 Table — (JPG) [file pone.0167235.s001.jpg]
